# Endoplasmic Reticulum Stress Pathway-Mediated Apoptosis in Macrophages Contributes to the Survival of *Mycobacterium tuberculosis*

**DOI:** 10.1371/journal.pone.0028531

**Published:** 2011-12-14

**Authors:** Yun-Ji Lim, Ji-Ae Choi, Hong-Hee Choi, Soo-Na Cho, Hwa-Jung Kim, Eun-Kyeong Jo, Jeong-Kyu Park, Chang-Hwa Song

**Affiliations:** 1 Department of Microbiology, College of Medicine, Chungnam National University, Daejeon, South Korea; 2 Infection Signaling Network Research Center, College of Medicine, Chungnam National University, Daejeon, South Korea; 3 Research Institute for Medical Sciences, College of Medicine, Chungnam National University, Daejeon, South Korea; Central Institute of Educational Technology, Canada

## Abstract

**Background:**

Apoptosis is thought to play a role in host defenses against intracellular pathogens, including *Mycobacterium tuberculosis* (Mtb), by preventing the release of intracellular components and the spread of mycobacterial infection. This study aims to investigate the role of endoplasmic reticulum (ER) stress mediated apoptosis in mycobacteria infected macrophages.

**Methodology/Principal Findings:**

Here, we demonstrate that ER stress-induced apoptosis is associated with Mtb H37Rv-induced cell death of Raw264.7 murine macrophages. We have shown that Mtb H37Rv induced apoptosis are involved in activation of caspase-12, which resides on the cytoplasmic district of the ER. Mtb infection increase levels of other ER stress indicators in a time-dependent manner. Phosphorylation of eIF2α was decreased gradually after Mtb H37Rv infection signifying that Mtb H37Rv infection may affect eIF2α phosphorylation in an attempt to survive within macrophages. Interestingly, the survival of mycobacteria in macrophages was enhanced by silencing CHOP expression. In contrast, survival rate of mycobacteria was reduced by phosphorylation of the eIF2α. Futhermore, the levels of ROS, NO or CHOP expression were significantly increased by live Mtb H37Rv compared to heat-killed Mtb H37Rv indicating that live Mtb H37Rv could induce ER stress response.

**Conclusion/Significance:**

These findings indicate that eIF2α/CHOP pathway may influence intracellular survival of Mtb H37Rv in macrophages and only live Mtb H37Rv can induce ER stress response. The data support the ER stress pathway plays an important role in the pathogenesis and persistence of mycobacteria.

## Introduction

Tuberculosis (TB) is a major problem despite current therapeutic regimens. The spread of tuberculosis is exacerbated by the development of multidrug-resistant strains of *Mycobacteria tuberculosis* (Mtb) infection [1]. Killing intracellular mycobacteria in MDR-TB patients and developing highly resistant therapeutic methods for treating TB patients are required to address this challenge. Mtb is one of the most successful human pathogens due to its ability to manipulate host cells via multiple pathways to achieve its survival.

Macrophages in the lungs are the first cells that defend against pathogen invasion and play an important role in the initiation and maintenance of immune responses against Mtb. Mycobacterial infection leads to the activation of multiple microbicidal mechanisms, such as phagolysosome fusion and respiratory burst, and the production of proinflammatory cytokines [2]. Macrophages infected with mycobacteria may undergo apoptosis to remove intracellular bacilli. Programmed cell death plays an important role in host responses against mycobacterial infection [3], [4]. The inhibition of host cell apoptosis by Mtb has been considered a potential virulence factor [5], [6]. However, the underlying mechanisms by which Mtb induces necrosis or inhibits apoptosis in macrophages are still largely unknown.

Recently we reported that mycobacterial antigen ESAT-6 induced ER stress-mediated apoptosis [7]. Within the ER, the unfolded protein response (UPR) control many secretory and cellular proteins and plays an important role in folding these molecules during their transit through the organelle [8]. There are a number of insults lead to protein misfolding in the ER such as nutrient deprivation, alterations in the oxidation-reduction balance, changes in calcium concentration, failure of post-translational modifications, or simply increases in secretory protein synthesis. The UPR alters the expression of ER chaperones to enhance the degradation of misfolded proteins. Additionally, the UPR inhibits protein synthesis to decrease the load within the ER [8]. For secretory proteins to fold properly and ensure survival, cells induce ER chaperone proteins to prevent the toxic accumulation of misfolded secretory proteins and ensure proper ER homeostasis [9]. A series of ER chaperones is involved in both the regulation of protein synthesis and the induction of cell death [10], [11]. Under prolonged ER stress, the UPR initiates signaling pathways that promote apoptosis. There are three ER-localized protein sensors: IRE1α (inositol-requiring 1α), PERK (double-stranded RNA-dependent protein kinase (PKR)-like ER kinase) and ATF6 (activating transcription factor 6) [12]. One of the components of the ER stress-induced apoptotic pathway is C/EBP homologous protein (CHOP) [13]. Another major UPR target protein is glucose-regulated protein 78 (GRP78/BiP), which plays an important role in protein folding and assembly, and targets misfolded proteins for degradation.

A recent study demonstrated that excess or misfolded proteins from *Saccharomyces cerevisiae* induce ER stress [14]. A central question of the present study is whether ER stress-induced apoptotic pathways play an important role in tuberculosis, because the ER stress pathway may be important in the overall control of cell viability [8], [10]. To contest this hypothesis, we measured induction of ER stress chaperones during mycobacterial infection, characterized ER stress-mediated apoptosis, and ascertained the effect of eIF2α/CHOP pathway on the survival of intracellular mycobacteria.

## Results

### Mtb infection induces programmed cell death and caspase activation

**Figure 1:**
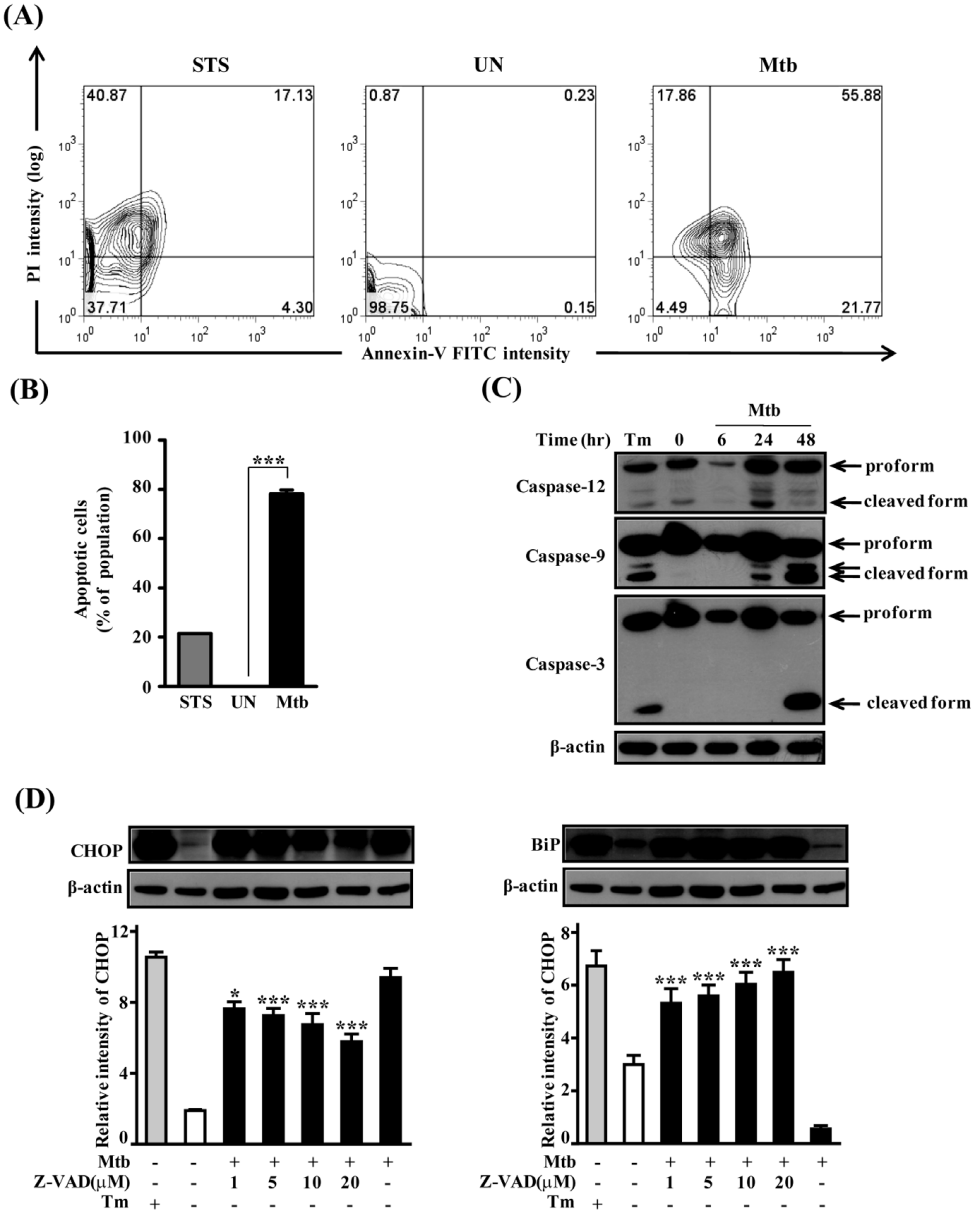
Mtb H37Rv infection induces apoptosis and caspase activation. (A) Raw264.7 cells were screened for induction of apoptosis using Annexin-V/PI staining after 48 h infection with Mtb H37Rv at a MOI of 1. Staurosporine (500 nM) was used for making positive control for apoptosis. After washing and Annexin V-/PI staining, cells were analyzed by flow cytometry. Data are respresentative of at least three independent experiments with similar results. (B) Quantitative analysis of the percentage of Annexin V-positive cells as described in B. ***, *P*<0.001 (C) Cellular levels of caspase-3, caspase-9, and caspase-12 during Mtb-infection in Raw264.7 cells. (D) The effect of caspase inhibitor z-VAD-fmk on the CHOP expression in Mtb-infected Raw264.7 cells. Raw264.7 cells were infected with Mtb at a MOI of 1 for 3 h, and then incubated for 0–48 h. The statistical significance (***P<0.001) of observed differences between z-VAD-fmk treated and untreated groups following infection with Mtb H37Rv were verified by two-tailed t-test. Immunoblot analysis was performed as described in Materials and Methods.

Apoptosis of macrophage plays important role in host defence against mycobacterial infections [15]. To investigate whether Mtb induced cytotoxicity was associated with the apoptotic pathway, flow cytometry was used to distinguish and quantitatively determine the percentage of dead, viable, apoptotic, and necrotic cells after Mtb infection (Fig. 1). The percentage of early apoptotic and late apoptotic cells was increased from 0.4% in unstimulated control cultures to 78.5% after Mtb infection. Since caspases are involved in ER stress-mediated cell death and are also activated by Mtb [16], [17], casapase activation was examined in Raw264.7 cells after Mtb infection. Procaspase-9 is known as a substrate of caspase-12 [18], which resides in the ER. Thus, we examined the effects of Mtb stimulation on activation of both caspase-9 and caspase-12. Caspase-9 and caspase-12 were activated in macrophages after Mtb infection and activated caspase-3 was strongly expressed at 48 h after Mtb infection (Fig. 1C). To further examine whether caspase activation is involved in CHOP expression, we cultured Raw264.7 cells infected with Mtb in the presence or absence of the broad-spectrum caspase inhibitor z-VAD-fmk (administered 1 h prior to infection). We found that z-VAD-fmk block Mtb-induced CHOP activation in macrophages, suggesting that the CHOP induction in macrophage infected with Mtb resulted primarily from capsase activation (Fig. 1D).

### ER sensor molecules are induced by Mtb infection

**Figure 2:**
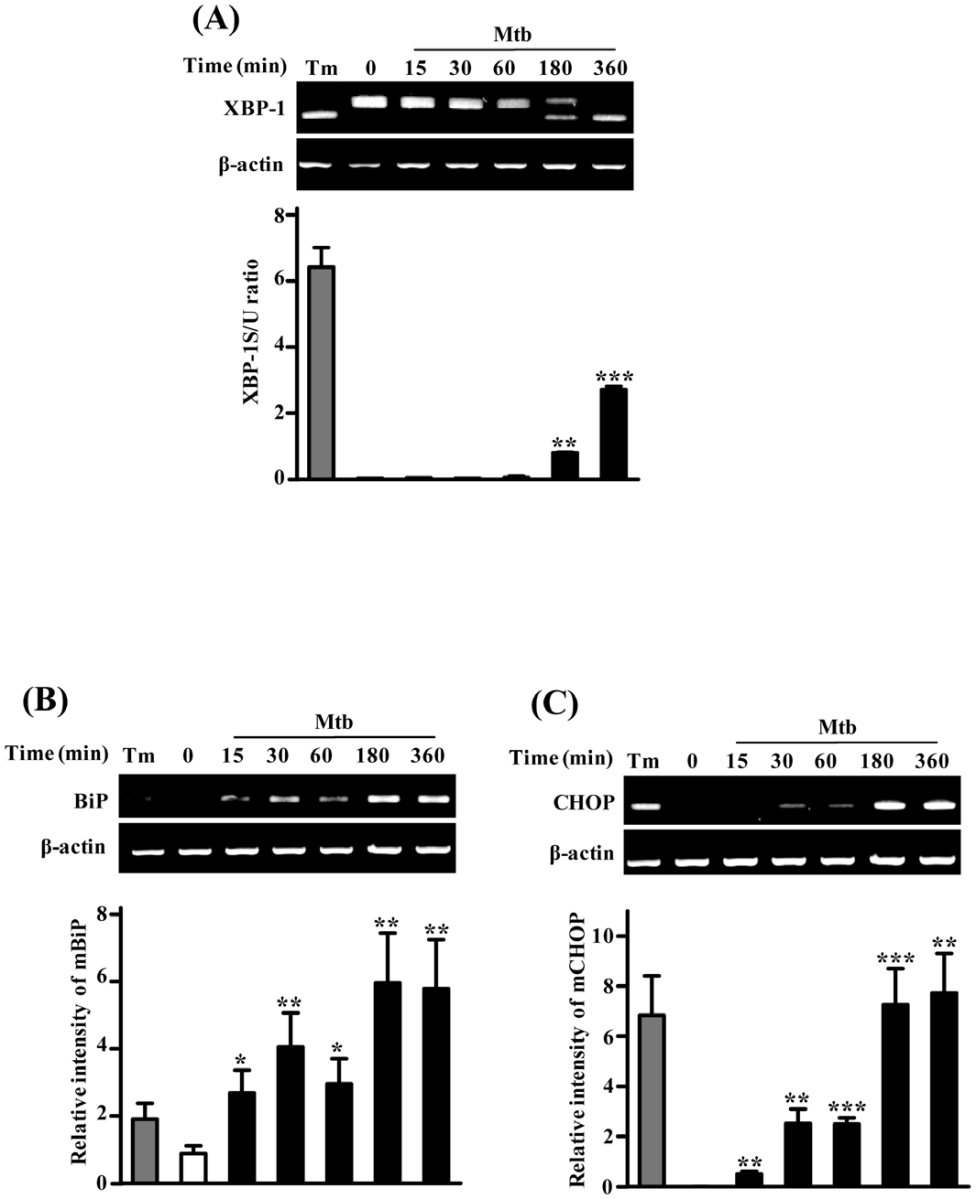
Time-course analysis mRNA for ER stress-related genes after Mtb H37Rv infection. Raw264.7 cells were infected with Mtb H37Rv (MOI = 1) for 3 h, and then incubated for 0–6 h. (A) XBP-1 mRNA splicing was determined by RT-PCR using specific primers that was used to amplify products of unspliced and spliced mRNA. The results represent the ratio of XBP-1 splicing to XBP-1 unsplicing (XBP-1S/U ratio). (B, C) PCR amplification with the primer pair corresponding to the BiP and CHOP mRNA was performed at the indicated time points. The results were quantified by densitometry. For a positive control, cells were treated with 2.5 µg/mL tunicamycin (Tm) for 6 h. The statistical significance (*P<0.05, **P<0.01 and ***P<0.001) of observed differences between Mtb H37Rv infected and uninfected groups were verified by two-tailed t-test. Representative data from three independent experiments are shown.

**Figure 3:**
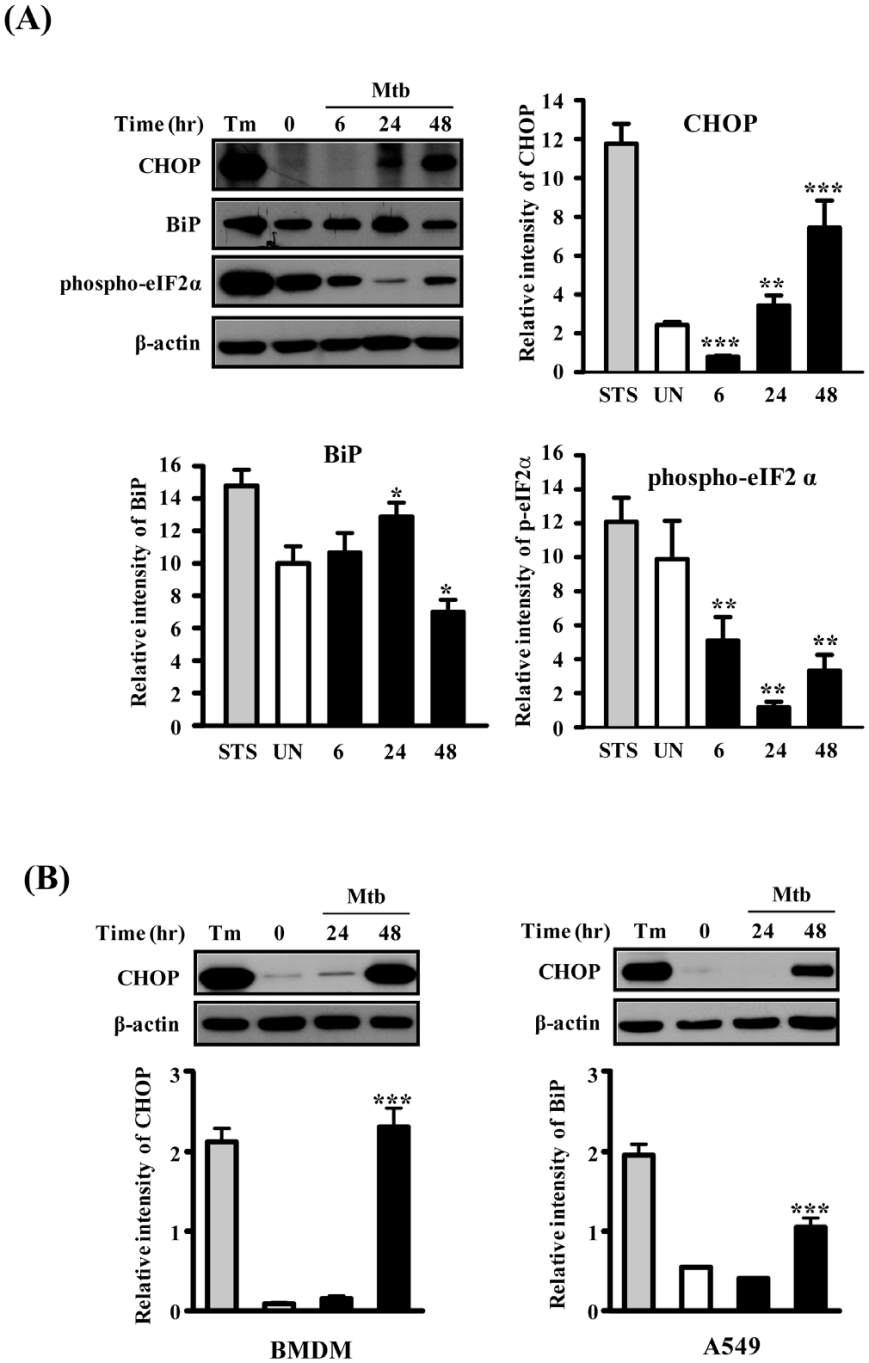
Mtb H37Rv infection induces ER stress sensor proteins in macrophage cells and CHOP is activated in BMDMs or A549 cells. Cells were infected with Mtb H37Rv (MOI = 1) for 3 h, and then incubated for 0–48 h. Immunoblot analysis was performed as described in Materials and Methods. The statistical significance (*P<0.05, **P<0.01 and ***P<0.001) of observed differences between Mtb H37Rv infected and uninfected groups were verified by two-tailed t-test. (A) Raw264.7 cells, (B) BMDMs or A549 cells. Representative data from three independent experiments are shown. STS: staurosporine.

The transcription factor CHOP is induced by ER stress and mediates ER stress-induced apoptosis [13]. To investigate other indicators of ER stress during Mtb infection, we examined splicing of XBP-1 indicative of IRE1 activation, BiP/GRP78, CHOP expression of both mRNA and protein levels in a time-dependent manner (Fig. 2 and 3). Expression of BiP and CHOP mRNA was gradually increased after Mtb infection and reached a maximum after 3 h. During ER stress, ER membrane-localized IRE1α is activated and the phosphorylated IRE1α catalyzes the splicing of XBP-1 mRNA [12]. The ratio of mXBP-1 splicing was markedly increased at 6 h after Mtb infection in Raw264.7 cells (Fig. 2).

At the protein level, CHOP and BiP were increased by Mtb infection. The induction of CHOP was increased from 24 h and peaked at 48 h after Mtb infection (Fig. 3). BiP expression was increased a little bit at 24 h and slightly decreased at 48 h, suggesting that BiP may operate to alleviate the ER stress to prevent apoptosis but prolonged ER stress may promote apoptosis through activation of CHOP. Since eIF2α phosphorylation has been suggested to be cytoprotective during ER stress [19], we examined phosphorylation of eIF2α by blotting the same membrane using an anti-phospho-eIF2α antibody. Interestingly, eIF2α phosphorylation was decreased gradually until 24 h after Mtb infection. Thus, these data indicate that Mtb infection may affect eIF2α phosphorylation in an attempt to survive within macrophages.

### The eIF2α-CHOP pathway may affect survival of mycobacteria in macrophages

**Figure 4:**
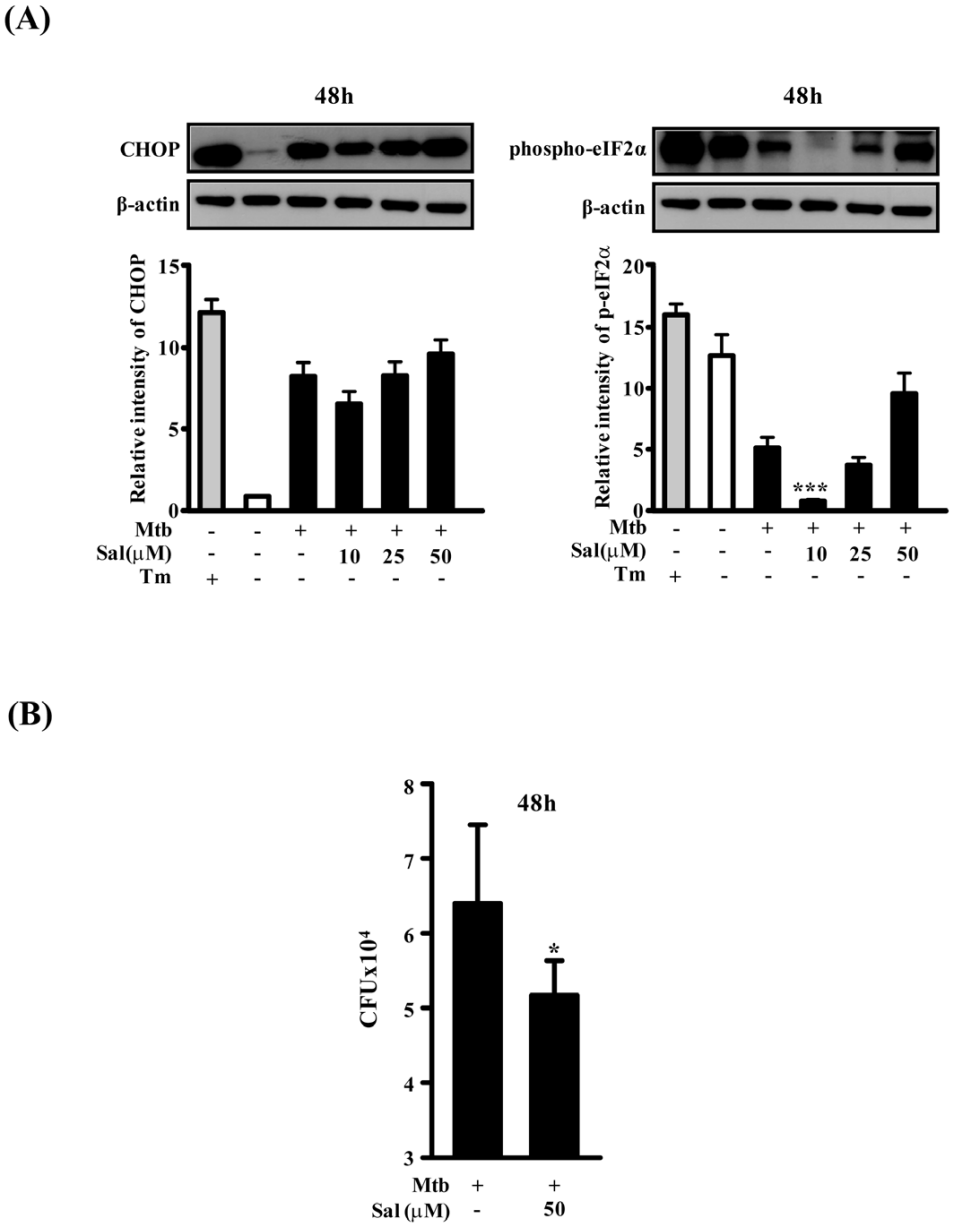
Effect of eIF2α phosphorylation on intracellular survival of Mtb H37Rv. Raw264.7 cells were pretreated for 30 min with indicated concentrations of salubrinal and then infected with Mtb H37Rv (MOI = 1) for 3 h. Salubrinal remained for the rest of the infection. (A) The cells were incubated for 48 h and Western blot analysis was performed using antibodies directed against CHOP, p-eIF2α and β-actin. DMSO alone was used as the negative control. (B) Quantification of intracellular survival of Mtb H37Rv in Raw264.7 cells pretreated for 30 min with salubrinal as described above. The cells were collected at 48 h postinfection with Mtb H37Rv and bacteria number was determined by CFU counting. The statistical significance (*P<0.05) of observed differences between salubrinal treated and untreated groups following infection with Mtb H37Rv were verified by two-tailed t-test. Data represent the mean±standard error of the mean (SEM) of values obtained in three independent experiments.

ER stress triggers apoptosis mainly through the PERK pathway via its downstream effectors phosphorylated eIF2α (p-eIF2α) and CHOP [9]. In figure 4, we show that p-eIF2α was decreased by Mtb infection. To identify the biological roles of eIF2α during Mtb infection, we used salubrinal, a selective inhibitor of eIF-2α that seems to target the PP1/GADD34 complex [20], for the determination of its effects on CHOP expression. The expression of CHOP and p-eIF2α was increased in response to salubrinal stimulation during Mtb infection.We expected that salubrinal treatment increased p-eIF2α and CHOP expression because salubrinal blocks eIF2αdephosphorylation. Interestingly, low dose of salubrinal treatment did not affect the phosphorylation status of eIF2α However, treatment with a high concentration of salubrinal (50 µM) induced phosphorylation of eIF2α (Fig. 4A). Similar expression pattern of CHOP protein was also observed. These data indicated that macrophage cells responded to Mtb infection by increasing p-eIF2α and CHOP levels, likely due to induction of the UPR. The decrease in eIF2α phosphorylation observed could result from resistance of mycobacteria to host translation inhibition.

To address the significance of p-eIF2α, we assessed the effects of salubrinal on the intracellular survival of Mtb in macrophages. We focused on the time point 48 h after Mtb infection because maximum CHOP production was observed. Interestingly, intracellular survival of Mtb was significantly decreased at a high concentration of salubrinal in Raw264.7 cells (Fig. 4B). These data provide evidence that ER stress-mediated apoptosis affects on intracellular survival of Mtb. The enhanced eIF2α phosphorylation in response to Mtb infection may elicit a proapoptotic response that is counteracted by Mtb under prolonged stress.

**Figure 5:**
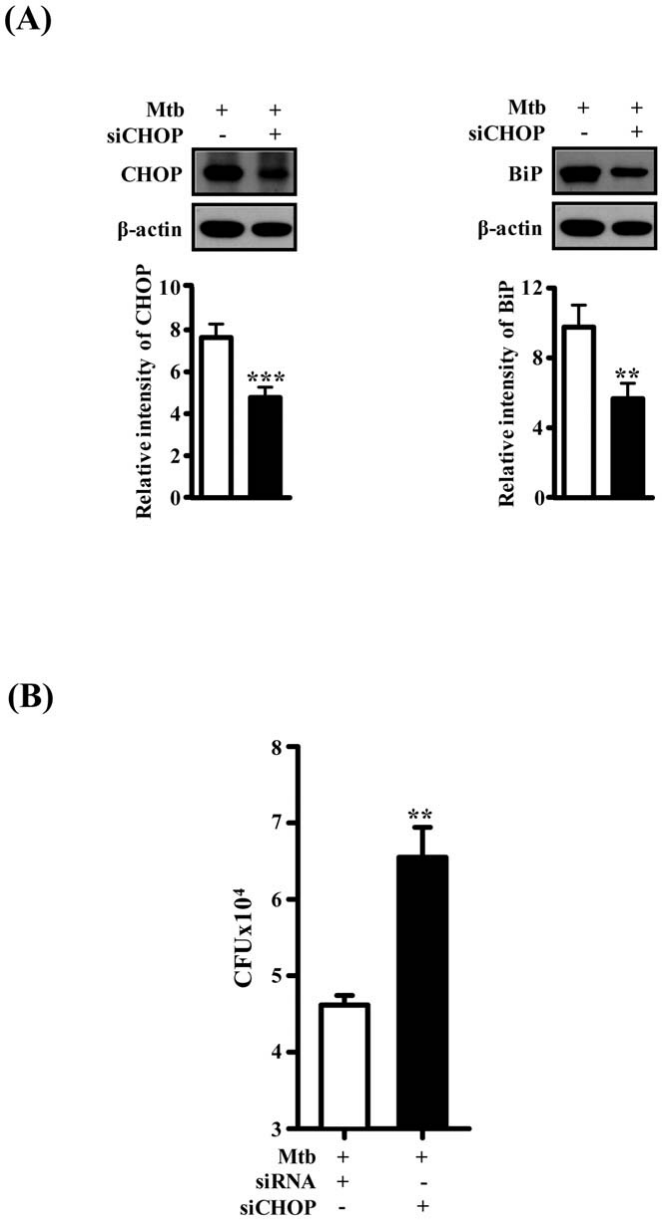
Effect of siCHOP on intracellular survival of Mtb H37Rv. (A) Representative images of Raw264.7 cells after transfection. Raw264.7 cells were transfected with siRNA (siControl, or siCHOP) for 5 h and then infected with Mtb H37Rv for 3 h. At 48 h after Mtb H37Rv infection, Western blot analysis was performed using antibodies directed against CHOP, BiP and β-actin. The experiments were repeated three times. (B) Quantification of intracellular survival of Mtb H37Rv in Raw264.7 cells pretreated with siRNA as described above. The cells were collected at 48 h postinfection with Mtb H37Rv and bacteria number was determined by CFU counting. The statistical significance (**P<0.01) of observed differences between siCHOP treated and untreated groups following infection with Mtb H37Rv were verified by two-tailed t-test. Data represent the mean ± SEM of values obtained in two independent experiments performed in triplicate.

To further investigate whether eIF2α/CHOP pathway is involved in intracellular survival of mycobacteria, we prepared CHOP siRNA to evaluate intracellular survival of Mtb. Raw264.7 cells were transfected with CHOP siRNA before Mtb infection. As shown in figure 5, transfection of CHOP siRNA resulted in Mtb infection-induced suppression, compared with cells transfected with control siRNA. As expected, intracellular survival of Mtb was increased in response to siCHOP. These data suggest that CHOP expression is important for controlling intracellular mycobacteria.

### Live mycobacteria induce ER stress molecules

**Figure 6:**
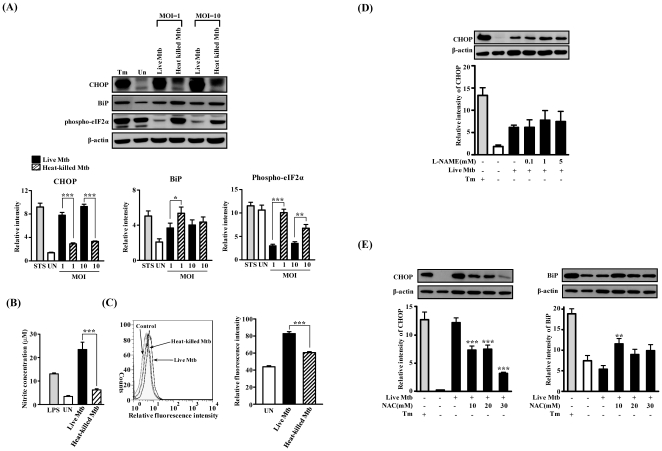
The expression of ER stress markers in Raw264.7 cells infected with live or heat-killed Mtb H37Rv. Raw264.7 cells were infected with live or heat killed Mtb H37Rv (MOI = 1 to 10) for 3 h, and then incubated for 48 h in the presence or absence of L-NAME or NAC. (A, D, E) Immunoblot analysis was performed as described in Materials and Methods. Representative data from three independent experiments are shown. (B) Effect of Mtb H37Rv infection on nitric oxide (NO) production was assessed indirectly by Griess reaction. (C) Representative flow cytometry histograms of superoxide at 48 h after Mtb H37Rv infection. Superoxide detection was evaluated by dihydroethidium (DHE) staining using flow cytometry. Data are means ± SEM of two independent experiments performed in triplicate. CHOP expression analysis after treatment with N-nitro-L-arginine methyl ester (L-NAME), a nitric oxide inhibitor (D) and N-acetyl-L-cysteine (NAC), a superoxide inhibitor (E). The statistical significance (*P<0.05, **P<0.01 and ***P<0.001) of observed differences between inhibitor treated and untreated groups following infection with Mtb H37Rv were verified by two-tailed t-test.

Since only live Mtb induced apoptosis when directly compared to the dead mycobacteria [21], we hypothesize that only live Mtb can induce ER stress responses. To examine whether dead bacteria could be responsible for inducing ER stress in macrophages, Raw264.7 cells were treated with live or heat-killed Mtb for 48 h. Cell lysates were examined for ER stress sensor molecules such as CHOP, BiP, and p-eIF2α. CHOP expression was induced by treatment with live, but not heat-killed, Mtb (Fig. 6). In contrast, BiP and p-eIF2α expression was increased in Raw264.7 cells infected with heat-killed Mtb compared with live Mtb. A similar pattern of ER stress sensors at a higher multiplicity of infection (MOI) was also observed with Mtb infection. These results suggest that ER stress induced CHOP expression may play an important role in anti-mycobacterial immunity.

Macrophages infected with Mtb generate reactive oxygen intermediates (ROI) and reactive nitrogen intermediates [22]. Since ER stress pathway involving CHOP is important in NO- and ROS-induced apoptosis [7], [23], we determined to investigate the production of NO and ROS from Raw264.7 cells infected with live Mtb and heat-killed Mtb (Fig. 6B, C). Interestingly, levels of NO were significantly higher in supernatants of live Mtb infected cells as compared to the supernatants from heat-killed Mtb infected cells (Fig. 6B). L-NAME, an inhibitor of nitric oxide synthase (NOS), inhibited NO production to a comparable extent in this experiment. However, produced NO does not affect on CHOP expression (Fig. 6D).

The production of superoxide was measured with dihydroethidium staining after Mtb infection. Live Mtb infected Raw264.7 cells produced significantly increased levels of ROS compared with heat-killed Mtb infected cells (Fig. 6C). The increased CHOP expression by Mtb infection was gradually decreased by ROS scavenger, NAC (Fig. 6E). These results suggest that ROS produced by Mtb infection may directly disturb ER function to activate CHOP expression even if NO production was different between live Mtb and heat-killed Mtb infected cells.

## Discussion

Macrophages infected with mycobacteria may undergo apoptosis to remove intracellular bacilli, whereas virulent mycobacteria prevent macrophage apoptosis to survive in cells [24]. It is important to understand the mechanisms of apoptosis regulation in the pathogenesis of tuberculosis.

Recent studies suggest that ER stress sensors are increased in granulomas in response to mycobacterial infection [25]. One of the components of the ER stress-mediated apoptosis pathway is CHOP [13]. However, there is not enough evidence to explain the role of ER stress in macrophages in tuberculosis.

Macrophage apoptosis induced by a high MOI-infection with virulent Mtb does not require TNF-α, caspases, or Toll signals [26], [27] but culminates in macrophage necrosis. The high MOI could promote extracellular spread of infection and formation of necrotic lesions in tuberculosis, while a low MOI infection leads to classical apoptosis [26], [27]. Here, we show that mycobacteria-infected macrophages undergo apoptosis at a low MOI, which is related to caspase activation. Induction of the ER stress-signaling pathway by Mtb infection has been confirmed by subsequent data showing that mRNA and protein levels of BiP and CHOP increase (Fig. 2, 3). BiP and CHOP are used as UPR markers for ER stress under pathological conditions [12]. Our results successfully showed upregulation of BiP and CHOP by Mtb infection (Fig. 3). BiP has been considered as a chaperone molecule that plays a key role in maintaining cell viability against various stressors [12], [28]. In contrast, CHOP is involved in ER stress-mediated apoptosis. Together, these data suggest that the earlier induced BiP could be upregulated for cell survival and later CHOP expression might be upregulated for apoptosis by a direct response to Mtb infection and releasing factors from Mtb-infected cells.

In the present study, we provided evidence that ER stress was induced by Mtb infection. We analyzed XBP-1 expression in Mtb infected macrophages because XBP-1 plays an important role in the regulation of innate immune response for host defense [29], [30]. We have shown that XBP-1 splicing is induced by Mtb infection (Fig. 1). Toll-like receptors (TLRs) play important roles in controlling Mtb intracellular replication and elimination [31]. Additionally, mycobacterial cell wall components can activate cells in a TLR-dependent manner [32]. The finding that macrophages under ER stress are hyper-responsive to TLR stimulation in an XBP-1-dependent manner supports our data that XBP-1 splicing is induced during Mtb infection [30]. Moreover, some candidate mRNA levels of regulated IRE1-dependent decay (RIDD) pathway (Heparan-α-glucosaminide N-acetyltransferase; HgNat, Biogenesis of lysosome-related organelles complex-1, subunit 1; Blos1, and Scavenger receptor class A, member 3; Scara3) were decreased after Mtb infection (data not shown), indicating that activated IRE1 during Mtb infection might degrade not only XBP-1 mRNA but also target mRNAs of RIDD pathway in response to ER stress. Thus, we suggest that TLR signaling may activate IRE1α and induce XBP-1 splicing during Mtb infection [32].

ER stress responses mediate the transient attenuation of mRNA translation by increasing eIF2α phosphorylation. Translation initiation factor eIF2α plays a key role in the regulation of protein synthesis in the ER [19]. Our results show that phosphorylation of eIF2α is decreased by Mtb infection (Fig. 3) and increased p-eIF2α by salubrinal treatment is correlated with CHOP induction (Fig. 4). Interestingly, increased p-eIF2α by salubrinal seems to reduce the intracellular survival after Mtb infection (Fig. 4). The phosphorylation of eIF2α at early time points post-infection was decreased after Mtb infection could be due to the loss of important functions that inhibit translation by Mtb in host cells. Similarly, respiratory syncytial virus (RSV) infection attenuates eIF2α phosphorylation to survive in hosts [33] and human papillomavirus E6 protein inhibits eIF2α phosphorylation to prevent PKR-mediated apoptosis [34]. Thus, it is possible that the p-eIF2α/CHOP pathway may control intracellular survival of Mtb and the ability to regulate the UPR in macrophages.

Caspase-12 is located in the ER and is responsible for ER-stress mediated apoptosis [35]. Processed caspase-12 activates caspase-9, followed by activation of caspase-3 [36]. Our study shows that caspase-12, caspase-9, and caspase-3 are activated by Mtb infection (Fig. 3), indicating that Mtb infection affected caspase-12 activation in macrophages. Activated caspase-12 initiates the proteolytic activity of other downstream caspases, including caspase-3. The importance of caspase-12 in immune responses has been reported, including dampening parasite clearance, inhibiting the production of proinflammatory cytokines, and bacterial clearance [37], [38], [39]. Thus, our results suggest that caspase-12 activation induced by the ER stress plays an important role in Mtb-infected macrophages.

CHOP is known as an inducer of apoptosis-favoring genes in response to ER stress. Because CHOP acts to repress Bcl-2 production and causes apoptosis, we hypothesized that through its role in ER stress-mediated apoptotic signaling, CHOP protein may cause macrophages to remove intracellular mycobacteria. Our data support an intimate link between CHOP expression and intracellular survival of Mtb from apoptosis due to ER stress responses (Fig. 5). Regulation of CHOP expression has been accepted as an approach to remove cancer cells through the induction of apoptosis [40], [41]. Although our data show that suppressed CHOP expression results in the increased survival of Mtb, it has yet to be determined whether the CHOP protein is critical in regulating mycobacteria in host cells. However, to our knowledge, there is no report that CHOP suppression affects intracellular survival of mycobacteria in macrophages.

Previously, we reported that ESAT-6, a protein secreted from Mtb, induced ER stress in human epithelial cells [7]. We hypothesize that immunogenic substances from Mtb or intracellular replication of Mtb can induce ER stress responses. In the current study, it is demonstrated that living Mtb induced CHOP expression, but heat-killed Mtb could not. Moreover, heat-killed Mtb induced stronger p-eIF2α than living Mtb, indicating living Mtb suppresses eIF2α phosphorylation. The suppressed p-eIF2α may allow mycobacteria to survive in the macrophage. Although the exact cause of ER stress in tuberculosis is still unclear, a recent paper showed that ER stress chaperones, including CHOP, were found in Mtb-induced granulomas [24]. Torres et al. [42] previously reported macrophages processed heat-killed Mtb more rapidly and efficiently than live Mtb. This finding may also explain why administration of heat-killed Mtb suppressed the ER stress response in macrophages. Our data suggest that living Mtb can induce ER stress-mediated apoptosis, but macrophages stimulated with heat-killed Mtb may overcome the UPR.

NO and ROS production is important to control of Mtb [22] and ER stress pathway are induced by NO or ROS [43], [44]. We have shown here live Mtb infection induces NO and ROS production in Raw264.7 cells. Interestingly, CHOP induction was decreased by NAC, an ROS scavenger, whereas treatment with NO scavenger had no effect. It can be postulated that produced NO is not enough to cause ER stress mediated apoptosis of macrophages during Mtb infection. Although the mechanisms of ROS activate ER stress pathway remains to be more investigated, these findings suggest that ROS-induced ER stress-mediated apoptosis is involved in the pathogenesis of tuberculosis.

We have demonstrated for the first time that live Mtb infection induces ROS productions and activates ER stress-mediated apoptosis. Specifically, regulation of the eIF2α/CHOP pathway plays an important role in intracellular survival of mycobacteria. Taken together, our observations reveal that ER stress pathway is one of the important components of host defense mechanisms against Mtb infection. Therefore, we suggest that the ER stress signaling pathway may be involved in Mtb-induced apoptosis to control intracellular growth of Mtb.

## Materials and Methods

### Mtb culture

*Mycobacterium tuberculosis* strain H37Rv (ATCC 27294) was grown in Middlebrook 7H9 liquid medium supplemented with 10% OADC (oleic acid, albumin, dextrose, catalase), 5% glycerol, and 0.05% Tween-80 and resuspended in phosphate-buffered saline (PBS) at a concentration of 1×10^8^ CFU/mL. Aliquots were frozen at −70°C until used. Heat-killed Mtb H37Rv was prepared by heating live H37Rv in PBS at 80°C for 30 min.

### Cell culture and Mtb infection

The murine macrophage cell line Raw264.7 cells and human lung adenocarcinoma epithelial cell line A549 (ATCC No. 185-CCL) were maintained in Dulbecco's modified Eagle's medium (DMEM) supplemented with 10% FBS, penicillin (100 IU/mL), and streptomycin (100 µg/mL). The cells (1×10^5^) were cultured in 12-well polypropylene tissue culture plates overnight at 37°C, 5% CO_2_ to allow cell adherence before infection. Bone marrow derived macrophages (BMDMs) were isolated from femurs and tibias of C57BL/6 mice (6–8 weeks old) and then differentiated by growth for 3–5 days in medium containing M-CSF (25 µg/mL; R&D). The cells were infected for 3 h with Mtb H37Rv (ATCC 27294) at a MOI of 1∶1∼10∶1. Then, cells were washed to remove noninfected bacteria and cultured with fresh complete medium without antibiotics and intracellular bacterial counts were determined at various times postinfection on Middlebrook 7H10 agar.

### Ethics Statement

All animal procedures were reviewed and approved by the Institutional Animal Care and Use Committee of Chungnam National University (Permit Number: 2010-2-32). All animal experiments were performed in accordance with Korean Food and Drug Administration (KFDA) guidelines.

### Reagents

Salubrinal, a selective inhibitor of eIF-2α, and tunicamycin (Tm, Calbiochem) were prepared as a concentrated stock solution (10 mg/mL) in dimethyl sulfoxide. N-nitro-L-arginine methyl ester (L-NAME, Sigma) and N-acetyl cysteine (NAC, Sigma) were dissolved in DMEM and diluted to the desired concentration directly in the culture medium. Raw264.7 cells were pretreated with indicated concentrations of salubrinal or NAC for 30 min before Mtb infection. DMSO alone was used as the negative control.

### RT-PCR analysis

Total RNA was prepared from the cultured Raw264.7 cells, reverse transcribed, and cDNA was used to amplify CHOP, BiP, and XBP-1, with β-actin as an internal control. All amplification reactions were performed as previously described [7].

### Immunoblotting analysis

Immunoblotting was performed as previously described [7]. The primary antibodies were anti-CHOP (Cell Signaling, MA), anti-GRP78/BiP (Cell Signaling), anti-phospho(Ser-51)-eIF2α (Assay Designs), anti-caspase-12 (Cell Signaling), anti-caspase-9 (Cell Signaling), anti-caspase-3 (Cell Signaling), and anti-β-actin (Santa Cruz Biotechnology). The secondary antibodies used in the study are goat anti-rabbit-IgG-HRP (Cell signaling), rabbit anti-mouse-IgG-HRP (Calbiochem). The blots were quantitated with a Gel Doc 2000 gel-documentation system (Bio-Rad). Actin is shown as a control for protein loading. As a positive control, tunicamycin (2.5 µg/mL) treated for 6 h

### Detection of nitric oxide (NO)

NO production was evaluated by nitrite accumulation in the supernatant using the Griess reaction. A portion (100 µL) of each culture supernatants was added to 100 µL of Griess reagent, and the absorbance at 540 nm was measured with a microplate reader.

### Reactive oxygen species (ROS)

The production of ROS was detected at 24 h post infection with Mtb (MOI = 1) by 20 µM dihydroethidium (DHE) staining for 30 min. Briefly, Raw264.7 cells were infected for 3 h with Mtb (MOI = 1). Cells were washed 3 times with Hanks' balanced salt solution, fixed with 4% paraformaldehyde and analyzed by FACSCanto II system (BD Biosciences, San Jose, CA, USA).

### Apoptosis analysis

Apoptotic cells were assessed by binding of Annexin V-FITC according to the manufacturer's instructions (BD Pharmingen, San Diego, CA). Binding of Annexin V-FITC and PI was analyzed by BD FACSCanto II flow cytometer (BD Biosciences) with FlowJo 7.6 software (Tree Star Inc).

### Gene silencing using small interfering RNA

Silencing of CHOP was achieved by the small interfering RNA (siRNA) technique. The siRNA (200 nM) for mouse CHOP mRNA target sequences (Bioneer Corporation, South Korea) and negative control siRNAs were purchased from Santa Cruz biotechnology, Inc. (Santa Cruz Biotechnology, Santa Cruz, CA). The siRNA oligonucleotides were transfected with into cultured Raw264.7 cells using Lipofectamine 2000 (Invitrogen, CA, USA) according to the manufacturer's instructions. After 5 h post-transfection, and the cells were cultured with fresh complete medium without antibiotics for infection and then harvested for western blotting or enumeration of intracellular bacteria.

### Statistical analysis

All experiments were done independently repeated at least three times. Statistical significance was tested at *P*<0.05 as critical value using student's t-test. Data are presented as the mean±95% confidence interval for mean.
